# Multi-modal multi-task internet sentiment recognition model based on mid-to-back fusion strategy

**DOI:** 10.1371/journal.pone.0362468

**Published:** 2026-11-10

**Authors:** Sichen Wu, Ruina Lin

**Affiliations:** Changsha University of Science and Technology, Changsha, China; International Information Technology University, KAZAKHSTAN

## Abstract

Previous studies of multimodal sentiment recognition have paid limited attention to the discrepancy between unimodal expression and multimodal perception in sentiment labeling, and to the question of where in the network the modalities should be fused. This paper proposes a multimodal multi-task sentiment recognition model based on a mid-to-back fusion strategy. Text, audio and visual inputs are encoded with the pre-trained BERT, Wav2Vec 2.0 and CLIP models, respectively, and refined by convolutional, recurrent and self-attention layers. Each unimodal branch is trained with its own unimodal sentiment label, so that modality-specific representations are optimized before fusion; the three high-level unimodal representations are then integrated by a residual multi-head self-attention module supervised by the multimodal label, and the four tasks are optimized jointly. On the Chinese CH-SIMS dataset the proposed model achieves a three-class accuracy of 70.14% and a binary accuracy of 75.09%, improving on the strongest published multi-task baseline by 1.1 and 3.4 percentage points, respectively; the binary-accuracy gain is statistically significant, with p < 0.01 in Welch’s t-test. Fusion-position ablations show that delaying fusion raises neutral-class accuracy from 28.87% with early fusion to 43.82% with mid-to-back fusion, and the model transfers to the English CMU-MOSI benchmark, on which it reaches a binary accuracy of 86.53%. The model still has room for improvement with respect to dataset diversity, the complexity of real-world applications, non-standard language and cross-cultural sentiment understanding. Overall, the proposed approach improves the effectiveness of multimodal fusion and offers a new perspective for multimodal sentiment recognition.

## 1 Introduction

Sentiment recognition, an important branch of human–computer interaction, has long been a focus of extensive research. Early studies concentrated on three unimodal channels: text, speech and vision. In the speech modality, research has covered the extraction of different acoustic features, the use of unlabeled data, the modeling of inter-relations in conversations, and the use of auxiliary tasks to improve generalization. Text-based research has explored the evolution of speakers’ sentiment in dialogues, the integration of global contextual information, speaker-specific effects, and the analysis of dialogue structure. Vision-based research has focused on spontaneous micro-expressions in video streams, the effect of facial occlusion on expression recognition, the transient nature of micro-expressions, and the estimation of expression intensity.

Although unimodal methods are effective in specific contexts, they remain limited in the comprehensiveness and granularity of sentiment recognition. Sentiment is rarely conveyed through a single channel; it emerges from the interaction of several modalities. To capture sentiment more accurately, researchers have therefore turned to multimodal sentiment recognition, in which cross-modal attention mechanisms, inter-modal complementarity and graph-based feature fusion are being investigated to improve robustness and generalization [1]. Despite this progress, how to fuse information from multiple modalities more effectively remains an open problem. To address it, we incorporate multi-task learning into our model; multi-task learning acts as a regularizer and has been widely applied in natural language processing (NLP).

For example, Li et al. [2] studied multi-task learning for spoken language understanding with overlapping slot sets, introduced a multi-task learning algorithm for slot-filling systems based on semi-Markov conditional random fields, and proposed a domain-based clustering method for automatically discovering shared slots. Collobert and Weston [3] used weight sharing in a convolutional neural network (CNN) for joint training across tasks and showed how multi-task and semi-supervised learning improve the generalization of shared tasks. Fully supervised deep multi-task models [4–6] have also achieved excellent performance. However, these approaches mostly used auxiliary tasks such as gender or speaker identity and did not adequately consider the differences between modalities. In this work we instead treat the three unimodal subtasks and the multimodal fusion subtask as the tasks of a multi-task framework: each unimodal subtask uses its own unimodal sentiment label, which explicitly accounts for the difference between unimodal expression and multimodal perception, while the fusion subtask uses the multimodal sentiment label.

Yu et al. [7] constructed CH-SIMS, a Chinese multimodal dataset with both unimodal and multimodal sentiment labels, and built a sentiment recognition model that treats the unimodal subtasks and the fusion subtask as a multi-task problem; Self-MM [8] later replaced the human unimodal labels with self-generated ones. These studies focused mainly on the effect of multi-task learning and did not systematically explore how different feature extractors and, in particular, different fusion positions affect the results. To address these issues, we propose a multimodal multi-task sentiment recognition model based on a mid-to-back fusion strategy.

Motivated by these limitations, this paper focuses on the discrepancy between unimodal expression and multimodal perception in the CH-SIMS setting and on how the fusion position interacts with multi-task learning. Unlike conventional mid-level fusion, which combines modalities immediately after feature extraction, and hybrid fusion schemes that mix early, mid-level and late interactions in a largely heuristic way, our approach first lets each unimodal branch learn a strong task-specific representation and prediction under its own labels, and then performs fusion at a later stage on high-level embeddings under a shared multimodal label. This design explicitly aligns three unimodal tasks with one multimodal task, so that the model preserves modality-specific cues while still exploiting cross-modal interactions.

The novelty of this work therefore lies not in any single component—pre-trained encoders, multi-task learning and attention-based fusion are all established techniques—but in how they are combined and where fusion takes place. First, in contrast to existing multi-task multimodal frameworks such as MLF-DNN, MTFN and MLMF [7] and Self-MM [8], which fuse either raw features or shallow unimodal features, the proposed model fuses only after each unimodal branch has been fully trained on its own unimodal labels, so that the fusion module receives task-optimized representations. Second, the fusion position is treated as an explicit design variable, and the same multi-task objective is evaluated at early, early–mid and mid-to-back positions, which isolates the effect of fusion timing from that of multi-task supervision. Third, modern self-supervised and contrastive pre-trained encoders, namely Wav2Vec 2.0 and CLIP, are compared with the hand-crafted features conventionally used on CH-SIMS within a single, controlled pipeline. In summary, the main contributions of this work are threefold. (1) We introduce a mid-to-back multimodal multi-task framework that explicitly models the gap between unimodal expression and multimodal perception by jointly optimizing three unimodal subtasks and one multimodal fusion task on CH-SIMS. (2) We conduct a systematic ablation study of feature extractors, comparing Wav2Vec 2.0, BERT and CLIP with Mel-spectrogram, OpenSMILE, Word2Vec, MediaPipe and OpenFace features, and of fusion positions, comparing early, early–mid and mid-to-back fusion, and show that mid-to-back fusion on top of strong unimodal branches yields the best trade-off between overall accuracy and neutral-class performance. (3) Experiments on CH-SIMS, supported by significance tests and confidence intervals, show that the proposed model outperforms several single-task and multi-task baselines and is competitive with recent Transformer-based methods on the three-class task; a confusion-matrix analysis further reveals that the design is particularly beneficial for the difficult neutral class, and a cross-dataset evaluation on CMU-MOSI indicates that the benefits transfer to an English benchmark.

## 2 Related work

### 2.1 Multimodal sentiment recognition

Multimodal sentiment recognition combines data from different modalities, such as text, speech and vision, to analyze comprehensively the sentiment expressed by a person. It generally involves two steps. The first is to mine the sentiment information carried by each modality and to extract modality-specific sentiment features. The second, and the key step, is to fuse the extracted features interactively so that the effective sentiment cues of every modality are fully expressed and exploited. Cheng et al. [9] introduced the information-bottleneck principle and constructed upper and lower bounds of mutual information from its objective in order to learn a concise and effective multimodal representation. Chen et al. [10] studied video sentiment analysis based on multimodal feature fusion. Han et al. [11] proposed MMIM, which hierarchically maximizes mutual information both between unimodal inputs and between the fused result and the unimodal representations, thereby reducing the loss of task-relevant information. Yang et al. [12] proposed a translation framework based on BERT (Bidirectional Encoder Representations from Transformers) for multimodal sentiment analysis.

More recently, Transformer-based architectures [13] have become the dominant paradigm for multimodal sentiment analysis [1]. The Multimodal Transformer (MulT) [14] introduced directional pairwise cross-modal attention to fuse unaligned sequences, and MISA [15] learns modality-invariant and modality-specific subspaces before fusion. Building on such cross-modal attention, EMT-DLFR [16] designs an efficient multimodal Transformer with dual-level feature restoration that remains robust when modality features are missing, and HyCon [17] applies hybrid intra- and inter-modal contrastive learning to tri-modal representations. Language-guided fusion has also proved effective: ALMT [18] learns an adaptive hyper-modality representation in which visual and acoustic features are filtered under the guidance of language features at several scales, and ConFEDE [19] decomposes each modality into text-centered similarity and dissimilarity features through contrastive learning. Vision–language pre-training such as CLIP (Contrastive Language–Image Pre-training) [20] provides visual embeddings that are already aligned with language and is increasingly used as a visual front-end in sentiment models. In parallel, large language models (LLMs) and multimodal LLMs are being explored for affective computing: Lian et al. [21] benchmarked GPT-4V on 21 emotion-recognition datasets in a zero-shot setting, and Emotion-LLaMA [22] instruction-tunes a multimodal LLM for emotion recognition and reasoning. On CH-SIMS specifically, recent work such as multi-layer feature fusion with multi-task learning [23] and the exchange-based adaptive multimodal Transformer AMTE [24] reports state-of-the-art binary accuracy. Most of these methods fuse the modalities at a single, fixed stage of the network and use the multimodal label as the only supervision; Self-MM [8] is an exception in that it generates unimodal labels automatically. Our work is complementary: it retains lightweight, frozen pre-trained encoders, uses the human-annotated unimodal labels of CH-SIMS as auxiliary supervision, and studies the fusion position itself as the main design variable.

Because the sentiment conveyed by different modalities of the same person during the same period may be inconsistent—for example, a speaker whose overall sentiment is positive may use positive words and tone while showing a negative facial expression—modalities can interfere with one another. The influence of each modality on the final recognition result therefore varies, which motivates a multimodal sentiment recognition model with strong recognition performance and good generalization.

### 2.2 Multi-task learning

Multi-task learning is a form of transfer learning in which several related tasks are trained simultaneously within one framework. It is suited to settings with many tasks whose features require a certain degree of generality, improves learning efficiency and has proved effective in multimodal tasks. Deep learning models usually require large numbers of training samples to reach high classification accuracy, but collecting such samples is time-consuming and labor-intensive. Multi-task learning is therefore an effective solution for learning several related tasks when the sample size is limited.

Multi-task learning nevertheless faces two major challenges during training: how to share network parameters and how to balance the learning of the different tasks. Zhao and Ju [25] used decoding modules to output logical tags within text and to extract entity relations from text, establishing an associated framework for multi-task learning. Wu and Ma [26] proposed a multi-task multimodal sentiment analysis model based on perceptual fusion with four tasks, namely multimodal, text, audio and image sentiment analysis, and a covariance-uncertainty loss that automatically weights the tasks; they concluded that the multi-task framework reduces noise and improves multimodal fusion. Li et al. [27] proposed a multi-task sentiment analysis method that integrates multi-head attention to extract important text features and dynamically categorizes multi-domain datasets during training; they built a multi-task sentiment classifier based on long short-term memory (LSTM) networks and point-wise convolution, designed a loss function for auxiliary classification tasks, and showed that multi-task learning with multi-head attention improves sentiment classification. By combining unimodal and multimodal tasks within multi-task learning, a model can learn the consistency and the differences between modalities more effectively and thereby improve accuracy.

### 2.3 Mid-to-back fusion strategy

Multimodal fusion strategies are commonly grouped by the stage at which the modalities are combined: early or feature-level fusion concatenates raw or low-level features, late or decision-level fusion combines unimodal predictions, and intermediate strategies fuse at hidden layers [1]. Mid-to-back fusion, also referred to as mid-to-late fusion, balances the depth of cross-modal interaction against the retention of modality-specific information. It fuses after unimodal processing has produced a high-level representation but before a final decision is made; that is, features or decision-related representations from different modalities are combined during the middle or later stages of the unimodal pipelines, so that both the richness of the unimodal features and the complementary information between modalities are exploited.

By combining multi-level processing, the mid-to-back fusion strategy strengthens the unimodal branches and at the same time exploits the complementary information across modalities, providing a more powerful and robust solution for a range of applications.

### 2.4 Pre-trained models

Pre-trained models are an application of transfer learning: they learn general representations from large corpora, which helps them perform downstream tasks more effectively. Because the dataset used in this study is relatively small, pre-training provides a good initialization, leading to better generalization and faster convergence on the target task.

Ding et al. [28] built a pre-trained model based on a bidirectional LSTM (Bi-LSTM) and showed that predictions with pre-trained models slightly outperform traditional machine-learning algorithms. Shen et al. [29] constructed text sentiment classifiers with TextCNN and BERT + Bi-GRU and obtained clear improvements over the baselines, demonstrating the advantage of pre-trained models in semantic feature learning. Zhou et al. [30] proposed a multi-task pre-trained BERT model that uses a large amount of unlabeled paragraph-level sentiment data and shares parameters across several classification tasks during pre-training, improving aspect-level sentiment classification. Lei et al. [31] used a Bi-LSTM to adjust the weights of each Transformer layer of a pre-trained model, dynamically fusing semantic representations of different granularity across layers to enhance the semantic representation space. Yang et al. [32] proposed a scene-transfer sentiment analysis model that integrates BERT pre-training and a Bi-LSTM: BERT extracts and represents word vectors, and a Bi-LSTM with attention captures long-distance dependencies and semantic features; experiments showed that the model outperforms traditional machine-learning and deep-learning models. In this work we adopt BERT [33] for text and the self-supervised Wav2Vec 2.0 model [34] for speech. In contrast to these unimodal pre-trained models, we use the cross-modal pre-trained model CLIP [20] for visual pre-processing. CLIP maps images and texts into a shared multimodal space and computes the cosine similarity between image and text vectors, so that text can be used to infer images. CLIP therefore strengthens the connection between modalities and, as our experiments show, provides substantial benefits to the downstream task.

## 3 Proposed model

In this study, we propose a multi-task multimodal sentiment recognition framework based on mid-to-back fusion, as shown in Fig 1. The framework integrates three modalities—visual, speech and text—through residual multi-head self-attention [13]. Each modality is first encoded by a frozen pre-trained model, namely CLIP, Wav2Vec 2.0 and BERT, respectively, refined by a modality-specific network and trained to predict its own unimodal sentiment label; the high-level unimodal representations are then fused and supervised by the multimodal label.

**Fig 1:**
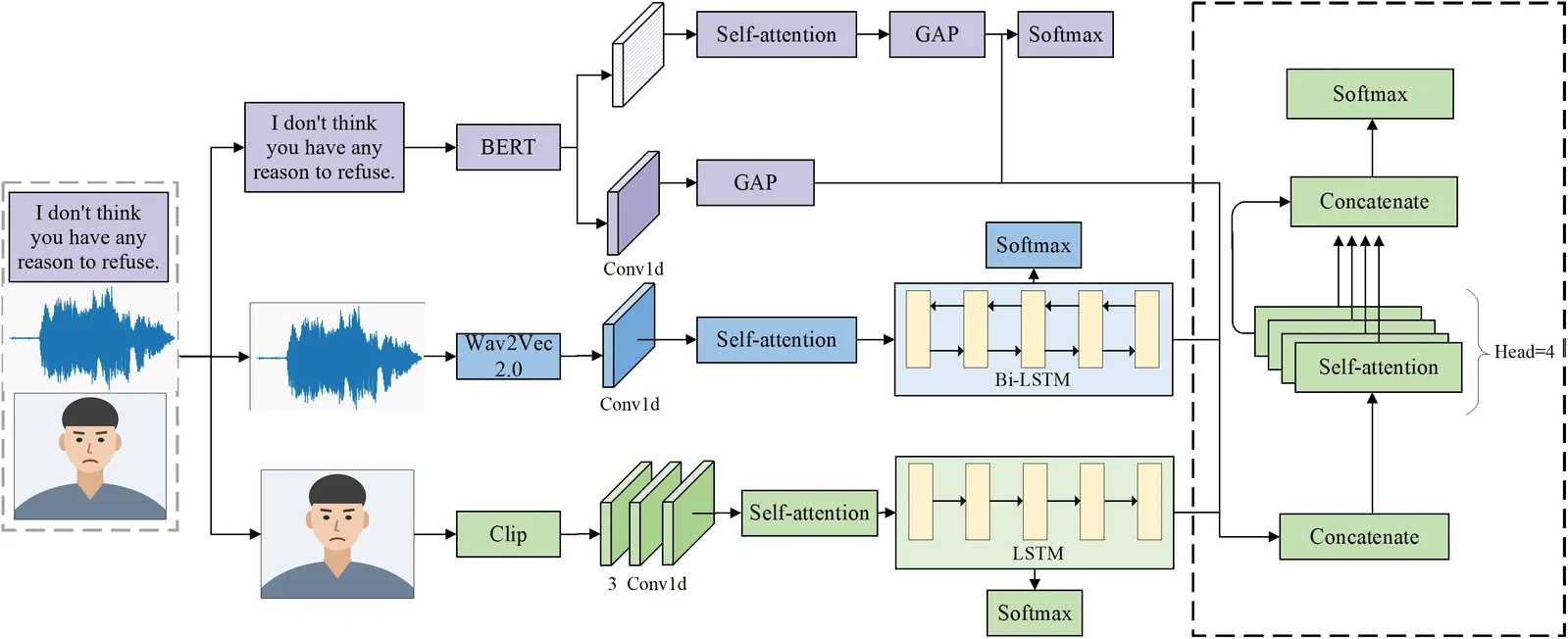
Multi-task sentiment recognition model based on the multimodal mid-to-back fusion strategy. The example input is a CH-SIMS segment whose utterance, translated into English, reads “I don't think you have any reason to refuse”. The face images are schematic drawings for illustrative purposes only and do not reproduce the original video frames. GAP: global average pooling; Conv1d: one-dimensional convolution; LSTM: long short-term memory; Bi-LSTM: bidirectional LSTM.

For the visual modality, considering the stability of human sentiment over short time intervals, the framework places an LSTM network after the convolution operations to learn the dynamic changes of facial image sequences over time. Through the self-attention mechanism, the model can effectively extract key information, which is particularly important for temporal sentiment analysis. This design not only improves data utilization but also adds a temporal dimension to the spatial dimension, enhancing the expressiveness of the model.

For the speech modality, after a convolution layer extracts preliminary features from the Wav2Vec 2.0 representations, a Bi-LSTM captures the fine-grained dynamics of the speech sequence. To enhance the analysis of speech, we also incorporate a self-attention mechanism, enabling the model to distinguish and extract subtle differences of sentiment expression within longer utterances.

For the text modality, we introduce not only an attention mechanism but also global average pooling (GAP), which avoids over-emphasizing local information at the expense of the overall semantics. For the text features used in fusion, we adopt a residual-like design in which the BERT output is processed along two parallel paths: the first path applies the attention mechanism and GAP described above, and the second path first extracts features by convolution and then applies GAP. The outputs of the two paths are concatenated, providing the model with a richer and more nuanced textual representation.

For the fusion strategy, we adopt mid-to-back fusion. Its core idea is to let each modality first build a high-level, task-optimized representation in its own branch and then to merge these representations in the later stage of the network, where a residual multi-head self-attention module learns cross-modal interactions before the final multimodal classifier. We also employ multi-task learning during training, optimizing four tasks concurrently. By sharing the underlying modality information, the unimodal branches and the fused branch reinforce and co-optimize each other during training, which improves training efficiency.

### 3.1 Visual modality prediction

For the visual modality, CLIP is used to extract frame-level visual embeddings $V_{x}$, which are further refined through a three-layer CNN. Each convolutional layer has 64 filters, with kernel sizes of 3, 1 and 3, respectively, and “same” padding preserves the temporal resolution:


$$\begin{array}{c} V_{C}=Conv1D_{\text{3}}\left(Conv1D_{\text{2}}\left(Conv1D_{\text{1}}\left(V_{x}\right)\right)\right) \end{array}$$ (1)

The extracted features are then fed into an LSTM to model temporal facial dynamics. Given input sequence $\{v_{t}\overset{T}{\underset{t=\text{1}}{\}}}$, the LSTM computes hidden states $h_{t}$ and cell states $c_{t}$ as:


$$\begin{array}{c} f_{t}=\sigma (W_{f}*[h_{t-1},x_{t}]+b_{f}) \end{array}$$ (2)

$$\begin{array}{c} i_{t}=\sigma (W_{i}*[h_{t-1},x_{t}]+b_{i}) \end{array}$$ (3)

$$\begin{array}{c} \overset{m}{\underset{t}{c}}=relu(W_{c}*[h_{t-1},x_{t}]+b_{c}) \end{array}$$ (4)

$$\begin{array}{c} c_{t}=f_{t}*c_{t}+i_{t}*\overset{m}{\underset{t}{c}} \end{array}$$ (5)

$$\begin{array}{c} o_{t}=\sigma (W_{o}*[h_{t-1},x_{t}]+b_{o}) \end{array}$$ (6)

$$\begin{array}{c} h_{t}=o_{t}*tanh\left(C_{t}\right) \end{array}$$ (7)

The final hidden output $h_{T}$ is:


$$\begin{array}{c} V_{LSTM}=LSTM\left(V_{c}\right) \end{array}$$ (8)

A softmax layer produces the unimodal visual sentiment prediction:


$$\begin{array}{c} V_{y}=softmax\left(V_{LSTM}\right) \end{array}$$ (9)

### 3.2 Speech modality prediction

Wav2Vec 2.0 features $A_{x}$ are processed through a 1D convolution followed by a self-attention layer:


$$\begin{array}{c} A_{c}=Conv1D\left(A_{x}\right) \end{array}$$ (10)

Self-attention is computed as:


$$\begin{array}{c} Attention\left(Q,K,V\right)=softmax\left(\frac{QK^{T}}{\sqrt{d_{k}}}\right)V \end{array}$$ (11)

yielding:


$$\begin{array}{c} A_{attn}=Attention\left(A_{c}\right) \end{array}$$ (12)

The enhanced features are then passed through a Bi-LSTM:


$$\begin{array}{c} A_{Bi-LSTM}=Bi-LSTM\left(A_{attn}\right) \end{array}$$ (13)

Concatenating both directions gives:


$$\begin{array}{c} A_{y}=softmax\left(A_{Bi-LSTM}\right) \end{array}$$ (14)

which is fed into a softmax layer to obtain the speech prediction $A_{y}$.

### 3.3 Text modality prediction

BERT features $T_{x}$ are refined using a 1D convolution and an attention mechanism:


$$\begin{array}{c} T_{c}=Conv1D\left(T_{x}\right) \end{array}$$ (15)

$$\begin{array}{c} T_{attn}=Attention\left(T_{c}\right) \end{array}$$ (16)

A global average pooling operation produces:


$$\begin{array}{c} T_{GAP}=GlobalAveragePooling1D\left(T_{attn}\right) \end{array}$$ (17)

The final text modality prediction is obtained as:


$$\begin{array}{c} T_{y}=softmax\left(T_{GAP}\right) \end{array}$$ (18)

### 3.4 Multimodal fusion

The fused multimodal representation $G_{x}$ is obtained by concatenating the outputs of the visual LSTM layer, the speech Bi-LSTM layer, the text global average-pooling layer, and the global average-pooled convolutional features $GlobalAveragePooling\text{1}D(T_{c})$.

This combined representation is then processed using a residual multi-head self-attention mechanism to capture cross-modal dependencies and enhance feature interaction.

(1) Linear projection: For each attention head i, the query, key, and value are obtained through separate linear projections:

$$\begin{array}{c} Q_{i}=G_{x}\overset{Q}{\underset{i}{W}},\text{\textbackslash{}hspace\{1em}\}K_{i}=G_{x}\overset{K}{\underset{i}{W}},\text{\textbackslash{}hspace\{1em}\}V_{i}=G_{x}\overset{V}{\underset{i}{W}} \end{array}$$ (19)

(2) Self-attention function: The attention weights and head output are computed using the standard scaled dot-product attention:

$$\begin{array}{c} \text{Attention}\left(Q_{i},K_{i},V_{i}\right)=\text{softmax}\left(\frac{Q_{i}\overset{T}{\underset{i}{K}}}{\sqrt{d_{k}}}\right)V_{i} \end{array}$$ (20)

where $d_{k}$ is the dimensionality of the key vectors used for scaling.

(3) Residual Attention: A residual connection is applied to stabilize optimization and preserve modality-specific information:

$$\begin{array}{c} ResAttention=Attention\left(Q_{i},K_{i},V_{i}\right)+Q_{i} \end{array}$$ (21)

(4) Multi-head merging: All heads are concatenated and projected to obtain the fused representation:

$$\begin{array}{c} {\text{G}}_{\text{attn}}=\text{Concat}({\text{head}}_{\text{1}},{\text{head}}_{\text{2}},\ldots ,{\text{head}}_{h})W^{O} \end{array}$$ (22)

where h is the number of heads and $W^{O}$ is the output projection matrix.

Finally, the multimodal prediction is generated by applying a softmax classification layer:


$$\begin{array}{c} G_{y}=softmax\left(G_{attn}\right) \end{array}$$ (23)

### 3.5 Multi-task learning objective

The overall multi-task objective consists of four tasks, namely three unimodal tasks and one fused task. The weighted joint loss is:


$$\begin{array}{c} Loss_{all}=\sum \gamma_{i}Loss_{i} \end{array}$$ (24)

Given datasets $D_{i}=\{(x_{ij},y_{ij})\}$ for each task $T_{i}$, the general multi-task objective is:


$$\begin{array}{c} {min}_{\theta ,\{\theta_{i}\overset{n}{\underset{i=1}{\}}}}\sum_{i=1}^{n}\lambda_{i}{ℒ}_{i}\left(\theta ,\theta_{i},D_{i}\right) \end{array}$$ (25)

where $\theta_{s}$ denotes shared parameters and $\theta_{i}$ denotes task-specific parameters.

### 3.6 Model complexity

The size of the network can be derived from the layer configuration of the released implementation. The pre-trained feature extractors BERT, Wav2Vec 2.0 and CLIP are frozen and used only once to pre-compute the input features, so they are excluded from the count. The three unimodal branches contain approximately 0.57 million parameters in total, of which about 0.15 M belong to the visual branch, 0.13 M to the speech branch and 0.30 M to the text branch; the text branch is the largest because its two convolutional layers operate on 768-dimensional BERT embeddings. The fusion module contains approximately 0.07 million parameters, so that the complete task network has about 0.64 million trainable parameters. The computational cost of the fusion module is dominated by the multi-head self-attention, whose time complexity is O(L^2^d) for a fused sequence of length L and width d = 64, followed by an LSTM layer of O(Ld^2^); with the sequence lengths used in this work this amounts to only a few million multiply–accumulate operations per sample, which is negligible compared with the cost of the pre-trained encoders. The model is therefore considerably lighter than end-to-end multimodal Transformers such as MulT [14] or ALMT [18], which fine-tune BERT and run cross-modal attention over the full modality sequences.

## 4 Materials and methods

### 4.1 Dataset selection

The dataset used in this study is CH-SIMS [7], a Chinese multimodal sentiment analysis dataset characterized by independent unimodal annotations. It contains 60 original videos divided into 2,281 video segments. Each segment has one multimodal annotation and three unimodal annotations, which ensures the diversity and depth of the labels.

During annotation, specific measures were taken to minimize interference between modalities: annotators were exposed only to the content of the modality they were annotating, which reduces cross-contamination of information between modalities. In terms of sentiment distribution, 54.3% of the 2,281 segments are labeled negative, 30.6% positive and 15.1% neutral. Following the official split, 1,368 segments form the training set, 456 the validation set and 457 the test set; the test set contains 248 negative, 140 positive and 69 neutral segments.

### 4.2 Multimodal feature extraction

(1) Feature extraction methods for the speech modality

For the speech modality, we compare three representative feature extraction pipelines: Mel-spectrograms, OpenSMILE and Wav2Vec 2.0. The Mel-spectrogram converts the waveform into a time–frequency matrix with 41 frames and 128 Mel bands, giving features of shape (41, 128). OpenSMILE summarizes hand-crafted prosodic and spectral descriptors such as pitch, energy and Mel-frequency cepstral coefficients (MFCCs), producing a sequence of 65-dimensional vectors with a final shape of (124, 65). Wav2Vec 2.0 [34] is a self-supervised representation learner that maps short audio windows to latent vectors; we use a Chinese fine-tuned XLSR-53 checkpoint and take the 512-dimensional outputs of its convolutional feature encoder, so that each utterance is represented as a sequence of 63 frames of 512-dimensional embeddings, that is, a matrix of shape (63, 512).

All three types of features are fed into the same downstream CNN–attention–Bi-LSTM classifier for a fair comparison. As reported in Table 5, Wav2Vec 2.0 achieves the best accuracy and weighted accuracy and is therefore adopted as the default speech feature extractor in the final model.

**Table 5:** Comparison of feature extraction methods for each modality with unimodal classifiers on the CH-SIMS test set. A, T and V denote the audio, text and visual modality; the best value per modality is in bold.

Modality	Feature extraction method	Acc	F1	Weighted Acc
A	Mel-Spectrogram	0.4995	0.5266	0.3893
	OpenSMILE	0.5295	**0.5488**	0.4661
	Wav2Vec 2.0	**0.5430**	0.5289	**0.4987**
T	Word2Vec	0.5836	0.5977	0.5025
	BERT	**0.6193**	**0.6955**	**0.5477**
V	MediaPipe	0.5450	0.5589	0.5058
	OpenFace	0.5827	0.6193	0.4998
	CLIP	**0.6031**	**0.7055**	**0.5775**

(2) Feature extraction methods for the text modality

For the text modality, we consider two widely used pre-trained language representations: Word2Vec and BERT [33], the latter in its bert-base-chinese version. With Word2Vec, each token is mapped to a 100-dimensional static embedding; after word segmentation each sentence becomes a matrix of size (sentence length, 100), and sentences are padded or truncated to 36 tokens, giving a feature tensor of shape (1368, 36, 100) for the training set.

With BERT, the input text is tokenized and embedded with token, segment and position embeddings and then processed by a multi-layer Transformer encoder. We take the contextualized token embeddings as text features, forming a tensor of shape (1368, 36, 768). These representations are then passed through a 1-D convolution, an attention layer and GAP in the text branch.

The results in Table 5 show that BERT features clearly outperform Word2Vec on all three metrics, namely accuracy, F1-score and weighted accuracy. BERT is therefore selected as the text feature extractor in the proposed framework.

(3) Feature extraction methods for the visual modality

For the visual modality, we compare MediaPipe facial-landmark features, OpenFace descriptors of gaze, head pose and facial action units and CLIP [20] image embeddings. For CLIP, 20 frames are sampled uniformly from each segment and encoded by the CLIP ViT-B/32 image encoder into 512-dimensional vectors, giving a feature matrix of shape (20, 512) per segment. All three feature types are fed into the same CNN–LSTM classifier. As Table 5 shows, CLIP features give the best accuracy, F1-score and weighted accuracy, and CLIP is therefore adopted as the visual feature extractor.

### 4.3 Model configuration

The framework of the proposed multimodal sentiment analysis model is shown in Fig 1. It comprises three modality-specific branches, for the visual, speech and text modalities, and a fusion module, whose configurations follow the released implementation.

In the visual branch, the CLIP frame features of shape (20, 512) pass through three one-dimensional convolutional layers, each with 64 filters, kernel sizes of 3, 1 and 3 and “same” padding, an LSTM layer with 64 units that captures the temporal dynamics of the frame sequence, and a fully connected softmax layer.

The speech branch consists of a one-dimensional convolutional layer with 64 filters, a kernel size of 3 and a stride of 2 applied to the Wav2Vec 2.0 sequence, a self-attention layer with 32 hidden units, a Bi-LSTM layer with 32 units per direction, giving a 64-dimensional output, and a fully connected softmax layer.

The text branch applies two parallel one-dimensional convolutional layers, each with 64 filters and a kernel size of 3, to the BERT token embeddings of shape (36, 768); one output serves as the query and the other as the value of an attention layer, and both the attended sequence and the query sequence are reduced by GAP to 64-dimensional vectors, which are concatenated into a 128-dimensional text representation before the fully connected softmax layer.

In the fusion module, the LSTM output of the visual branch, the Bi-LSTM output of the speech branch and the two GAP outputs of the text branch are concatenated to form a 256-dimensional representation, which is processed by a residual multi-head self-attention layer with 4 heads and a key dimension of 32, followed by a 64-unit projection and the multimodal softmax classifier; a dropout rate of 0.2 is applied to improve robustness.

Training uses a multi-task learning strategy with loss weights of 0.3, 0.3, 0.3 and 1 for the visual, speech, text and fusion tasks, respectively, which assigns a higher priority to the multimodal fusion task. We use a batch size of 32, 30 training epochs and the Adam optimizer with an initial learning rate of 0.001.

Implementation details. The model is implemented in Python 3.8 with TensorFlow 2.9.3 and Keras 2.9 and trained on a single NVIDIA GeForce RTX 3060 Laptop GPU with 6 GB of memory under CUDA 11. The pre-trained features are extracted once with PyTorch and the Hugging Face and CLIP libraries: the text features are the 768-dimensional token embeddings of bert-base-chinese; the speech features are the 512-dimensional outputs of the convolutional feature encoder of a Chinese fine-tuned XLSR-53 Wav2Vec 2.0 checkpoint, wav2vec2-large-xlsr-53-chinese-zh-cn, computed at 16 kHz; and the visual features are the 512-dimensional embeddings of the CLIP ViT-B/32 image encoder applied to uniformly sampled frames. These features are kept frozen, so the unimodal branches and the fusion module are the only trainable parts, with about 0.64 million parameters in total as detailed in Section 3.6. Extracting the features for all 2,281 segments takes about 110 min in total on this machine: approximately 2 min for the BERT text features, 45 min for the Wav2Vec 2.0 speech features and 60 min for the CLIP frame features, the latter because every frame of a segment is encoded before temporal sampling. This is a one-off cost, after which training the fusion stage takes 3–4 s per epoch of 43 mini-batches of 32 segments, that is, about two minutes for a full 30-epoch run. No explicit early stopping is applied; the checkpoint with the lowest validation loss is retained and used for all reported results. Because the unimodal encoders are frozen, the fusion head converges within the first few epochs: the training loss decreases steadily while the validation loss reaches its minimum early and then rises slightly, indicating mild over-fitting that is mitigated by dropout and by best-checkpoint selection. Hyper-parameters were chosen by a small grid search on the validation set over the fusion hidden dimension {32, 64, 128}, the dropout rate {0.1, 0.2, 0.3} and the number of attention heads {2, 4, 8}, as reported in Section 5.5; the unimodal loss weights were selected from three candidate settings, 0.4/0.4/0.7, 0.4/0.5/0.3 and 0.3/0.3/0.3, with the fusion weight fixed at 1. Random seeds are not fixed; instead, because a run is inexpensive, every reported number of the proposed model is the mean ± standard deviation (SD) over 12 independent training runs with different random initializations, and the run-to-run SD is small: Acc-3 = 70.14 ± 0.80.

### 4.4 Ethics statement

The individual in this manuscript has given written informed consent (as outlined in PLOS consent form) to publish these case details.

## 5 Experiments and evaluation

### 5.1 Baseline models

The proposed multimodal multi-task sentiment recognition model based on the mid-to-back fusion strategy is compared with the following existing multimodal sentiment recognition models to validate its effectiveness.

For fairness and transparency, we clarify the provenance of the baseline results. All baselines are evaluated on the same dataset and the same training/validation/test split as our model. Results for the standard CH-SIMS benchmark models are quoted from the CH-SIMS benchmark [7] and the corresponding original publications, using their reported values, including variance where available, whereas all results of the proposed model are obtained under identical conditions and reported as mean ± SD over 12 independent runs.

(1) Early Fusion LSTM (EF-LSTM) [35] first concatenates the initial inputs of the three modalities and then uses an LSTM to capture long-range dependencies in the sequence.
(2) Late Fusion DNN (LF-DNN) [7], in contrast to EF-LSTM, first learns unimodal features and then concatenates them before classification.
(3) Memory Fusion Network (MFN) [36] accounts for view-specific and cross-view interactions, models them continuously through a special attention mechanism and summarizes them over time with a multi-view gated memory. MFN requires word-level alignment across the three modalities.
(4) Low-rank Multimodal Fusion (LMF) [37] performs efficient multimodal fusion with modality-specific low-rank factors, capturing cross-modal interactions at a much lower cost than full tensor fusion.
(5) Tensor Fusion Network (TFN) [38] explicitly models view-specific and cross-view dynamics by constructing a multi-dimensional tensor that captures unimodal, bimodal and trimodal interactions across the three modalities.
(6) Multimodal Transformer (MulT) [14] uses directional pairwise cross-modal attention to enable interactions between multimodal sequences across different time steps and to adapt the stream from one modality to another.
(7) Multi-task variants. MLF-DNN, MLMF and MTFN [7] are the multi-task counterparts of LF-DNN, LMF and TFN provided with the CH-SIMS benchmark; they add the three unimodal labels as auxiliary supervision and are therefore the most directly comparable baselines for our multi-task model. They are marked with an asterisk in Table 2.

**Table 2:** Accuracy in percent of the multi-task model compared with published multi-task baselines on CH-SIMS. Values are mean ± SD; the best value in each column is in bold. * Multi-task variants provided with the CH-SIMS benchmark [7].

Model	Acc-3	Acc-2
MLF-DNN*	69.06 ± 3.1	71.65 ± 1.2
MLMF*	67.70 ± 2.2	64.02 ± 1.8
MTFN*	69.02 ± 0.3	69.05 ± 0.8
**MLMER (ours)**	**70.14 ± 0.8**	**75.09 ± 0.5**

### 5.2 Evaluation criteria and statistical analysis

For the comparisons of single-task versus multi-task learning, feature extraction methods and fusion positions, we use accuracy, F1-score and weighted accuracy as evaluation metrics. Accuracy is the percentage of correct predictions among all predictions; a higher value indicates better performance. The F1-score is the harmonic mean of precision and recall and reflects the reliability of the predictions; for example, a model with high precision but low recall is likely to be affected by class imbalance, so a higher F1-score indicates a more balanced model. Weighted accuracy averages the per-class accuracies weighted by the class distribution; comparing it with accuracy reveals how much the accuracy is influenced by the sample distribution. For the comparison with published baselines we report the multi-class accuracy Acc-k with k ∈ {2, 3}, where Acc-3 is the accuracy of the negative/neutral/positive classification and Acc-2 the binary-classification accuracy, following the CH-SIMS protocol [7].

Statistical analysis. Because each configuration is trained several times, we treat the per-run test scores as samples and compare the proposed model with each baseline using Welch’s two-sample t-test, which does not assume equal variances [39]; two-sided p-values below 0.05 are considered significant. We additionally report the difference in mean accuracy together with its 95% confidence interval (CI), computed from the t-distribution with Welch–Satterthwaite degrees of freedom. The proposed models are evaluated over 12 independent runs, as described in Section 4.3. For the published baselines, whose per-run scores are not available, the tests are computed from the published mean and SD with n = 5, the number of seeds used by the CH-SIMS benchmark; these results should therefore be read as approximate.

### 5.3 Comparison with baselines

Tables 1 and 2 report Acc-3 and Acc-2 for the single-task and multi-task settings, respectively; the best value in each column is shown in bold, and Table 3 gives the corresponding statistical tests.

**Table 1:** Accuracy in percent of the single-task model compared with published single-task baselines on CH-SIMS. Values are mean ± SD; the best value in each column is in bold.

Model	Acc-3	Acc-2
EF-LSTM	51.73 ± 2.0	55.28 ± 2.4
MFN	63.89 ± 1.9	62.11 ± 1.5
MulT	65.03 ± 2.1	67.03 ± 2.2
LF-DNN	66.91 ± 1.2	67.22 ± 0.5
LMF	64.38 ± 2.1	62.91 ± 1.2
TFN	64.46 ± 1.7	66.01 ± 1.6
**MLSER (ours)**	**68.87 ± 1.8**	**72.91 ± 0.8**

**Table 3:** Statistical comparison of the proposed models with the baselines of Tables 1 and 2. Each entry gives the difference in mean accuracy in percentage points with its 95% CI, followed by the two-sided p-value of Welch’s t-test; n = 12 runs for the proposed models and n = 5 runs with published SDs for the baselines.

Comparison	ΔAcc-3 [95% CI]	p (Acc-3)	ΔAcc-2 [95% CI]	p (Acc-2)
MLSER vs EF-LSTM	+17.14 [14.68, 19.60]	< 0.001	+17.63 [14.68, 20.58]	< 0.001
MLSER vs MFN	+4.98 [2.64, 7.32]	0.001	+10.80 [8.97, 12.63]	< 0.001
MLSER vs MulT	+3.84 [1.27, 6.41]	0.010	+5.88 [3.18, 8.58]	0.003
MLSER vs LF-DNN	+1.96 [0.32, 3.60]	0.023	+5.69 [4.99, 6.39]	< 0.001
MLSER vs LMF	+4.49 [1.92, 7.06]	0.005	+10.00 [8.54, 11.46]	< 0.001
MLSER vs TFN	+4.41 [2.29, 6.53]	0.001	+6.90 [4.95, 8.85]	< 0.001
MLMER vs MLF-DNN*	+1.08 [−2.74, 4.90]	0.483	+3.44 [1.97, 4.91]	0.002
MLMER vs MLMF*	+2.44 [−0.26, 5.14]	0.067	+11.07 [8.85, 13.29]	< 0.001
MLMER vs MTFN*	+1.12 [0.55, 1.69]	< 0.001	+6.04 [5.07, 7.01]	< 0.001
MLMER vs MLSER (multi- vs single-task)	+1.27 [0.06, 2.48]	0.041	+2.18 [1.61, 2.75]	< 0.001

(1) Single-task accuracy comparison

In the single-task setting we compare existing models with the proposed single-task variant, denoted MLSER, on the binary and three-class tasks. As Table 1 shows, MLSER achieves the highest accuracy on both tasks, improving on the strongest baseline, LF-DNN, by 1.96 percentage points on Acc-3, with a 95% CI of 0.32 to 3.60 and p = 0.023, and by 5.69 points on Acc-2, with a 95% CI of 4.99 to 6.39 and p < 0.001. The improvements over all six baselines are significant on both metrics, with p ≤ 0.023, as detailed in Table 3. MLSER also exhibits small run-to-run fluctuation, indicating stable behavior.

(2) Multi-task accuracy comparison

In the multi-task setting we compare the proposed multi-task model, denoted MLMER, with the multi-task baselines of the CH-SIMS benchmark. As Table 2 shows, MLMER obtains the best Acc-3 and Acc-2. On the three-class task its advantage over the strongest baseline, MLF-DNN, is modest, + 1.08 points with a 95% CI of −2.74 to 4.90 and p = 0.48, and not statistically significant owing to the large variance of MLF-DNN, whose SD is 3.1; the advantage over MTFN, + 1.12 points, is significant with p < 0.001, and that over MLMF, + 2.44 points, is marginal with p = 0.067. On the binary task, in contrast, MLMER improves on all three baselines by 3.44 to 11.07 points, and every difference is significant with p ≤ 0.002, as detailed in Table 3. MLMER also has the smallest SD among the multi-task models. Comparing Tables 1 and 2, multi-task supervision raises the accuracy of our own architecture from 68.87% to 70.14% on Acc-3, with a 95% CI of 0.06 to 2.48 and p = 0.041, and from 72.91% to 75.09% on Acc-2, with a 95% CI of 1.61 to 2.75 and p < 0.001, so the benefit of the unimodal auxiliary labels is significant on both tasks and clearest on the binary task.

To assess whether the proposed mid-to-back fusion and multi-task design generalize beyond the Chinese CH-SIMS dataset, we additionally evaluated MLMER on the English multimodal sentiment benchmark CMU-MOSI [40] under the standard binary-classification protocol. Because CMU-MOSI provides only a multimodal label, all four outputs, that is, the three unimodal branches and the fusion branch, are supervised with the multimodal label in this experiment, following the same procedure as in the released code. MLMER attains an Acc-2 of 86.53% and an F1 of 86.62%, which is competitive with or superior to representative baselines such as TFN, LMF, MulT and Self-MM; the strong Self-MM baseline, for example, reaches 85.98% Acc-2 [8]. These results indicate that the advantages of the proposed approach are not specific to CH-SIMS but transfer to a different language and dataset. Extending the evaluation to CMU-MOSEI, MELD and IEMOCAP, which differ in label granularity and conversational structure, is left for future work, as discussed in Sect 6.4.

(3) Comparison with recent Transformer-based methods

To position the proposed model with respect to recent Transformer-based approaches, Table 4 lists the three-class accuracy on CH-SIMS reported for representative methods published between 2017 and 2025, quoted from the original publications or from the unified M-SENA benchmark [41] as reproduced in [18]. We restrict this comparison to Acc-3 because the binary protocol differs between publications, the M-SENA benchmark, for instance, distinguishing negative from non-negative, whereas the three-class task is defined identically. Note also that these methods use the official CH-SIMS features, namely BERT text embeddings, hand-crafted acoustic features and OpenFace visual features, and were not re-trained by us, so the comparison is indicative rather than strictly controlled.

**Table 4:** Three-class accuracy in percent on CH-SIMS of recent methods as reported in the literature, compared with the proposed model.

Method	Year	Fusion paradigm	Acc-3 (%)	Source
TFN [38]	2017	Tensor fusion	65.12	[18]
LMF [37]	2018	Low-rank tensor fusion	64.68	[18]
MulT [14]	2019	Cross-modal Transformer	64.77	[18]
Self-MM [8]	2021	Self-supervised multi-task	65.47	[18]
ALMT [18]	2023	Language-guided hyper-modality Transformer	68.93	[18]
Cai et al. [23]	2025	Multi-layer fusion + multi-task	67.77	[23]
MLMER (ours)	–	Mid-to-back fusion + multi-task	**70.14 ± 0.80**	This work

As Table 4 shows, MLMER attains a higher Acc-3 than the recent language-guided Transformer ALMT, at 68.93%, and the 2025 multi-task model of Cai et al., at 67.77%, as well as the earlier tensor- and Transformer-based methods, at 64.7–65.5%. Part of this margin is likely attributable to the stronger CLIP and Wav2Vec 2.0 front-ends shown in Table 5, and a fully controlled comparison in which all methods share the same features remains future work; nevertheless, the result indicates that a comparatively lightweight mid-to-back fusion network with unimodal auxiliary supervision is competitive with considerably larger cross-modal Transformers on the three-class task.

### 5.4 Ablation studies and error analysis

This section presents ablation studies of the feature extractors and of the fusion position, and analyzes the error patterns of the model.

(1) Comparison of feature extraction methods for each modality

Table 5 compares the feature extraction methods for each modality. For the speech modality, Wav2Vec 2.0 achieves the highest accuracy and the highest weighted accuracy, indicating that it predicts well across the different classes. OpenSMILE has a slight advantage in F1-score, suggesting a higher recall in some cases, but the difference from Wav2Vec 2.0 is small. Considering all metrics, Wav2Vec 2.0 is selected as the speech feature extractor.

For the text modality, BERT clearly outperforms Word2Vec in accuracy, F1-score and weighted accuracy. The higher accuracy shows that BERT classifies more correctly, the large increase in F1-score reflects a better balance between precision and recall, and the higher weighted accuracy confirms that BERT maintains its performance across classes with different frequencies. BERT is therefore selected for the text modality.

For the visual modality, CLIP leads MediaPipe and OpenFace by a clear margin in accuracy, F1-score and weighted accuracy, indicating that it handles different types of data while maintaining high precision and recall. CLIP is therefore selected as the visual feature extractor.

On the basis of these comparisons, Wav2Vec 2.0, BERT and CLIP are identified as the best feature extractors for their respective modalities and are used in all subsequent multimodal experiments.

In addition, we compared different network architectures for each modality, as illustrated in Fig 2.

**Fig 2:**
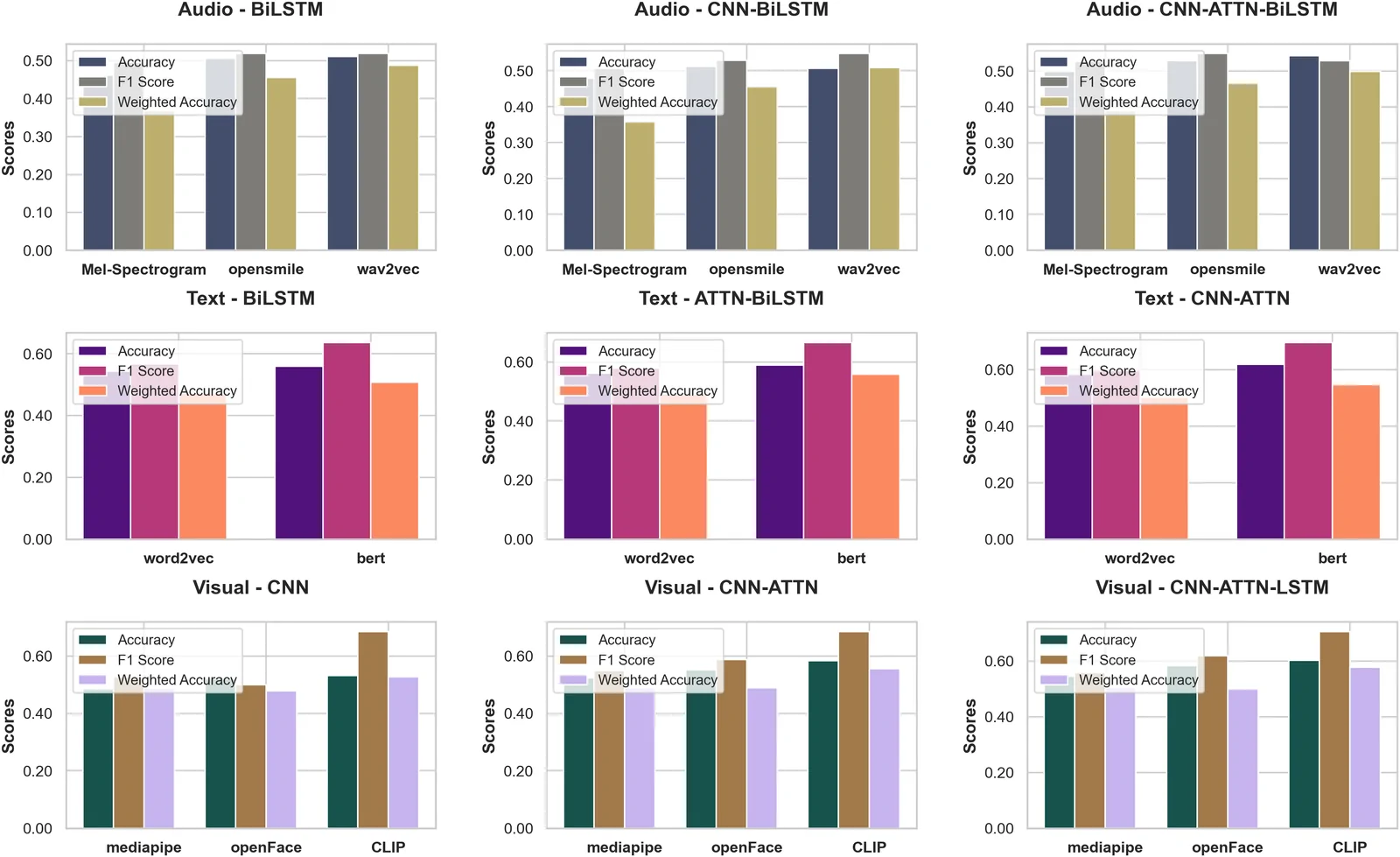
Performance comparison of unimodal models with different network architectures and feature extractors. Rows correspond to the audio, text and visual modality; bars show accuracy, F1-score and weighted accuracy.

For the audio modality we compared Bi-LSTM, CNN–Bi-LSTM and CNN–attention–Bi-LSTM architectures with Mel-spectrogram, OpenSMILE and Wav2Vec 2.0 features. The CNN–attention–Bi-LSTM architecture performed best with every feature type; with Wav2Vec 2.0 features it reached the highest accuracy, 0.5430, an F1-score of 0.5289 and a weighted accuracy of 0.4987, a clear improvement over the Bi-LSTM and CNN–Bi-LSTM models. These results highlight the capability of the CNN–attention–Bi-LSTM architecture for audio and the importance of Wav2Vec 2.0 features for audio sentiment classification.

For the text modality we compared Bi-LSTM, attention–Bi-LSTM and CNN–attention architectures with Word2Vec and BERT features. The CNN–attention model with BERT features performed best, with an accuracy of 0.6193, an F1-score of 0.6955 and a weighted accuracy of 0.5477, emphasizing the role of the CNN–attention structure when it is combined with BERT features.

For the visual modality we compared CNN, CNN–attention and CNN–attention–LSTM architectures with MediaPipe, OpenFace and CLIP features. The CNN–attention–LSTM model with CLIP features performed best, with an accuracy of 0.6031, an F1-score of 0.7055 and a weighted accuracy of 0.5775, the highest values in all visual experiments. CLIP features yield particularly large gains when combined with the more advanced architectures, confirming their importance for visual sentiment recognition.

(2) Comparison of fusion positions in the single-task and multi-task settings

To investigate the effect of the fusion position, we measured the accuracy of models fused at different positions in both the single-task and the multi-task setting (Table 6).

**Table 6:** Acc-3 in percent of different fusion positions in the single-task and multi-task settings. Values are mean ± SD over 12 runs; the best value is in bold.

No.	Single-task		Multi-task	
Fusion positions		Fusion positions	Accuracy
1	Early fusion	59.43 ± 0.72	Early fusion	61.72 ± 2.1
2	Early–mid fusion	63.89 ± 1.2	Early–mid fusion	65.02 ± 1.4
3	Mid-to-back fusion	**68.87 ± 1.8**	Mid-to-back fusion	**70.14 ± 0.8**

This experiment serves as an ablation of the fusion mechanism: early fusion concatenates the pre-trained features of the three modalities before any modality-specific processing; early–mid fusion fuses the outputs of the first convolutional layer of each branch; and mid-to-back fusion fuses the high-level outputs of the LSTM, Bi-LSTM and attention/GAP layers as described in Section 3.4. The same fusion module and the same training protocol are used in all three cases.

The results show a clear trend: in both the single-task and the multi-task setting, placing the fusion at a later stage of the network, that is, mid-to-back fusion, markedly improves classification accuracy. Under multi-task learning, mid-to-back fusion outperforms early–mid fusion by 5.12 points, with a 95% CI of 4.14 to 6.10, and early fusion by 8.42 points, with a 95% CI of 7.03 to 9.81, both with p < 0.001; the corresponding gains in the single-task setting are 4.98 and 9.44 points, again with p < 0.001. Moreover, at every fusion position the multi-task model is more accurate than its single-task counterpart, and at the two later positions it is also less variable. These findings highlight the advantage of late-stage fusion and provide an empirical basis for selecting fusion strategies in multimodal systems.

To analyze the effect of the fusion position further, we visualized the confusion matrices of the three fusion positions in Fig 3, where (a) is early fusion, (b) early–mid fusion and (c) mid-to-back fusion.

**Fig 3:**
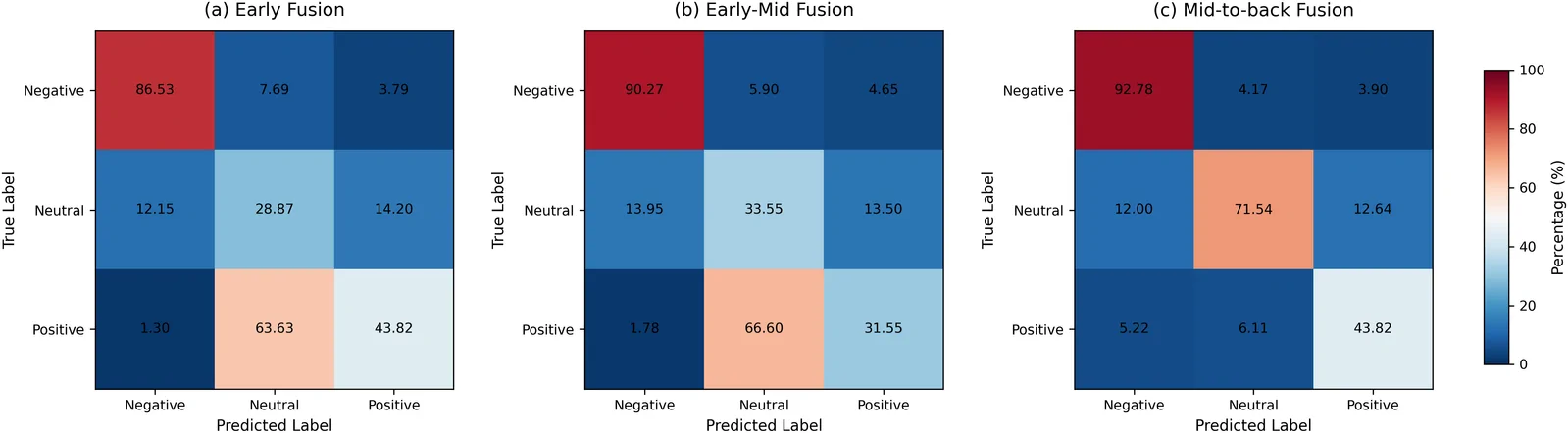
Row-normalized confusion matrices, in percent, of the multi-task model at different fusion positions: (a) early fusion, (b) early–mid fusion, (c) mid-to-back fusion.

The early-fusion model recognizes the negative class well, with 86.53% accuracy, but is less accurate for the positive class, at 63.63%, and worst for the neutral class, at 28.87%, indicating a high sensitivity to negative sentiment but difficulty with positive and especially neutral expressions. Many neutral expressions have low arousal and weak acoustic or visual cues, so they are easily confused with the positive or negative class.

With early–mid fusion the accuracy rises to 90.27% for the negative class, 66.60% for the positive class and 33.55% for the neutral class. Compared with early fusion, the negative and positive classes improve, the negative class markedly so, but neutral recognition remains difficult despite a small gain. This is mainly because cross-modal inconsistencies, for example neutral text with negative prosody or a flat facial expression, increase the ambiguity of neutral samples.

Mid-to-back fusion improves the model further: the accuracy reaches 92.78% for the negative class, 71.54% for the positive class and 43.82% for the neutral class. This strategy is the most accurate for all three classes, with the largest gain for the neutral class. The improvement is attributable to the fact that each modality first builds a stronger modality-specific representation before fusion, which better preserves the subtle cues indicative of neutrality.

Error analysis. To quantify the difficulty of neutral-class recognition, we computed the class-wise precision, recall and F1-score of the proposed model on the test set. The neutral class obtains by far the lowest recall, and most misclassified neutral segments are assigned to the negative class rather than to the positive class, which is consistent with the low-arousal, weak-cue nature of many neutral segments and with the predominance of the negative class in CH-SIMS, which accounts for 54.3% of all segments against 15.1% for the neutral class. Inspection of the misclassified test segments reveals two recurrent patterns. The first consists of short, factual utterances such as questions, statements of fact or instructions, whose text, audio and visual annotations are all neutral; lacking any polarity cue, they are drawn towards the majority negative class. The second consists of segments with conflicting modalities, for example a lexically positive utterance delivered with a negative tone or facial expression, or a complaint delivered cheerfully, where the unimodal branches disagree and the fused prediction follows the stronger non-neutral cue. Neutral recognition therefore remains the main open problem on CH-SIMS, as also observed for the benchmark baselines in [7].

In summary, across early, early–mid and mid-to-back fusion the overall accuracy increases consistently as fusion is delayed, and the neutral-class accuracy rises from 28.87% to 33.55% and 43.82%, indicating that postponing fusion allows the model to preserve modality-specific cues that are informative for ambiguous or low-arousal expressions. This result highlights the advantage of the mid-to-back strategy, especially for the neutral class.

### 5.5 Hyper-parameter sensitivity

To assess whether the performance of the proposed model depends on a finely tuned configuration, we conducted a sensitivity analysis of the three principal hyper-parameters of the fusion module, namely the hidden dimension, the dropout rate and the number of attention heads. Adopting a one-factor-at-a-time protocol, each hyper-parameter is varied over a set of representative values while the other two are fixed at their default settings. The resulting Acc-2, Acc-3 and F1 are reported as mean ± SD over 12 independent runs.

As shown in Table 7, the model is largely insensitive to these hyper-parameters within the examined settings. For the fusion hidden dimension, Acc-3 increases from 68.62% to 70.14% as the dimension grows from 32 to 64, whereas a further increase to 128 brings no additional improvement, with 69.03%; a dimension of 64 thus provides a favorable balance between representational capacity and over-fitting risk. The number of attention heads follows a comparable trend, peaking at four heads with an Acc-3 of 70.14% and decreasing only marginally when the number of heads is halved, to 69.62%, or doubled, to 69.18%, which indicates that the multi-head fusion module is not highly sensitive to this setting. For the dropout rate, the default value of 0.2 yields the highest Acc-3 and F1 and a near-best Acc-2, whereas a smaller rate of 0.1 or a larger rate of 0.3 results in only slight changes. The use of a moderate dropout rate is also consistent with the mild over-fitting of the fusion head observed in Section 4.3. Overall, Acc-3 varies by less than two percentage points across all examined settings, and the default configuration, namely hidden dimension 64, dropout 0.2 and four attention heads, consistently attains the best or near-best results on all three metrics. Together with the small run-to-run SDs, these observations confirm that the reported performance is robust to both hyper-parameter choices and random initialization, rather than being the product of extensive hyper-parameter tuning.

**Table 7:** Hyper-parameter sensitivity analysis. Values are mean ± SD over 12 runs; the default setting is in bold.

Hyper-parameter	Value	Acc-2 (%)	Acc-3 (%)	F1 (%)
	32	73.84 ± 0.91	68.62 ± 0.76	64.83 ± 1.02
Fusion hidden dim	**64 (default)**	**75.09 ± 0.50**	**70.14 ± 0.80**	**66.12 ± 0.73**
	128	73.97 ± 1.15	69.03 ± 1.06	65.47 ± 1.21
	0.1	75.22 ± 0.96	69.58 ± 0.87	65.74 ± 0.99
Dropout rate	**0.2 (default)**	**75.09 ± 0.50**	**70.14 ± 0.80**	**66.12 ± 0.73**
	0.3	73.68 ± 1.12	68.96 ± 1.01	64.52 ± 1.26
	2	74.72 ± 0.84	69.62 ± 0.77	65.78 ± 0.82
Attention heads	**4 (default)**	**75.09 ± 0.50**	**70.14 ± 0.80**	**66.12 ± 0.73**
	8	73.76 ± 1.08	69.18 ± 1.03	65.22 ± 1.10

## 6 Analysis and discussion

### 6.1 Impact of the feature extraction method of each modality

(1) Speech modality

For the speech modality we compared Mel-spectrogram, OpenSMILE and Wav2Vec 2.0 features with a common network architecture, and Wav2Vec 2.0 performed best. This is attributable to its design: Wav2Vec 2.0 [34] is a self-supervised convolutional–Transformer model pre-trained on large amounts of unlabeled speech with a contrastive objective, in which the model must identify the true quantized latent representation of a masked time step among distractors, a task closely related to noise-contrastive estimation (NCE). Through this pre-training, Wav2Vec 2.0 captures high-level speech representations that surpass traditional frame-based features and provide strong inputs for the downstream task.

(2) Text modality

For the text modality we compared Word2Vec and BERT with the same network structure, and BERT outperformed Word2Vec. The fundamental reason is that Word2Vec assigns a single static vector to each word and therefore cannot capture polysemy or context-dependent meaning, whereas BERT [33] uses a bidirectional Transformer encoder that considers the context before and after each word, producing a dynamic representation of the same word in different contexts. Through pre-training, BERT learns deep bidirectional relations within language and adjusts word vectors according to context, effectively addressing the polysemy problem of Word2Vec.

(3) Visual modality

For the visual modality we compared three feature extraction methods with a common architecture, and CLIP performed best. This advantage is attributable to CLIP [20] being a contrastive multimodal pre-trained model trained on a large collection of image–text pairs. Unlike conventional image recognition models, CLIP does not merely learn to identify individual objects but captures the holistic content of an image, including the objects, their relative positions and their semantic and logical relations. This comprehensive learning strategy allows CLIP to transfer to a variety of vision tasks with strong generalization and high performance.

### 6.2 Impact of the fusion position on sentiment recognition

(1) Single-task learning

In the single-task setting we fused at three positions: early, early–mid and mid-to-back. Mid-to-back fusion was the most effective. This can be attributed to the fact that each modality performs feature extraction independently before the mid-to-back stage, so that each branch fully exploits its intrinsic information; fusing afterwards maximizes the advantage of multimodality and extracts richer information from the fused features for more accurate classification.

(2) Multi-task learning

In the multi-task setting the same three fusion positions were compared, and mid-to-back fusion again performed best, for the same reason: each modality conducts in-depth feature extraction through its own network before fusion, and the unimodal auxiliary tasks further encourage each branch to produce sentiment-relevant representations. The multi-task model is more accurate than the single-task model at every fusion position, which indicates that in complex multimodal settings the combination of a late fusion stage with unimodal auxiliary supervision is an effective way to strengthen classification.

Moreover, compared with early and early–mid fusion, mid-to-back fusion yields a relative improvement for the neutral class, although neutral sentiment still lags behind positive and negative sentiment in recognition performance. This observation is consistent with the intrinsic ambiguity of neutral expressions and confirms that a more informative fusion strategy can partially mitigate, but not eliminate, the difficulty of neutral-sentiment classification.

### 6.3 Modality importance analysis

To examine whether the three modalities contribute equally to the fused prediction—that is, their relative importance—we analyze the attention-weight distribution of the fusion module, which complements the modality-ablation analysis, in which text alone reaches an Acc-3 of 65.19%, text and audio together 67.54%, and all three modalities 70.14%. Aggregating the multi-head self-attention weights over the test set, the average attention received by each modality is 0.41 for text, 0.36 for visual and 0.23 for audio, a clearly non-uniform distribution in which text is the most attended modality and audio the least. This importance ordering is consistent with the ablation results: every modality contributes and the full tri-modal model performs best, with an Acc-3 of 70.14%. Although the visual modality receives more attention than the audio modality, removing the audio modality causes a comparable accuracy drop, indicating that audio provides complementary prosodic cues that are less redundant with text. The ablation further shows that performance degrades gracefully under missing modalities, with any single modality already well above chance and any two modalities recovering most of the performance. Overall, the modalities do not contribute equally, which supports explicitly modeling the discrepancy between unimodal expression and multimodal perception. We note that attention weights are only an indirect indicator of importance; in future work we plan to employ more rigorous attribution methods such as SHAP [42], Integrated Gradients [43] or perturbation-based analysis to quantify the contribution of each modality and of individual time steps to the fused prediction.

### 6.4 Discussion

Because CH-SIMS provides independent unimodal annotations in addition to the multimodal label, it contains samples in which the unimodal labels disagree with one another or with the multimodal label. Such cases are precisely what the proposed multi-task, mid-to-back fusion design targets: by first learning strong modality-specific representations under unimodal supervision and fusing only at a later stage, the model preserves modality-specific cues that early fusion would otherwise suppress when the modalities convey inconsistent signals. The gains of the multimodal model over the unimodal models indicate that the model benefits from this complementary, and sometimes conflicting, information.

We further note that the observed gains can be attributed to the proposed architecture rather than to dataset-specific properties alone. The fusion-position ablation over early, early–mid and mid-to-back fusion is conducted on the same dataset and features, so the consistent improvement obtained by moving fusion towards later layers reflects the benefit of the mid-to-back strategy itself, and the cross-dataset evaluation on CMU-MOSI shows that the approach generalizes beyond CH-SIMS. At the same time, the multi-task formulation relies on datasets that provide independent unimodal annotations, so part of the multi-task benefit is naturally associated with the availability of such annotations.

Limitations. Several limitations should be acknowledged. First, the multi-task formulation relies on human-annotated unimodal labels, which are available in CH-SIMS but rare in other benchmarks; on datasets without such labels the auxiliary tasks must use the multimodal label, as in our CMU-MOSI experiment, or self-generated labels as in Self-MM [8]. Second, our evaluation covers one Chinese and one English dataset; claims about cross-cultural sentiment understanding, and about conversational corpora such as MELD and IEMOCAP that involve dialogue context and categorical emotions rather than sentiment polarity, therefore remain to be tested experimentally. Third, the comparison with recent Transformer-based methods in Table 4 relies on published numbers obtained with different feature front-ends. Fourth, the significance tests against the published baselines are computed from their published means and SDs rather than from per-run scores, and the Acc-3 advantage over the strongest multi-task baseline, MLF-DNN, is not statistically significant. Finally, the neutral class remains difficult, and the class imbalance of CH-SIMS, with only 15.1% neutral segments, probably contributes to this.

## 7 Conclusion

In this study we introduced MLMER, a multimodal multi-task sentiment recognition model based on a mid-to-back fusion strategy. Each unimodal branch is optimized under its own sentiment label before fusion, and the high-level representations are then fused through residual multi-head self-attention and supervised by the multimodal label. On CH-SIMS, MLMER outperforms the single-task and multi-task baselines considered, with statistically significant gains on the binary task, achieves a three-class accuracy that compares favorably with recent Transformer-based methods, and substantially improves neutral-class recognition; the design also transfers to CMU-MOSI. In future work we plan to explore more advanced architectures and more effective auxiliary tasks, to evaluate the model on additional benchmarks such as CMU-MOSEI, MELD and IEMOCAP, to conduct controlled comparisons with recent methods under identical features, and to employ rigorous attribution methods such as SHAP, Integrated Gradients and perturbation-based analysis to explain the fused predictions.

The present model operates at the segment level, consistent with the annotation granularity of CH-SIMS. Extending it to longer conversations or continuous streams, in which sentiment evolves over time, is a promising direction: the model could be applied in a sliding-window manner with an additional temporal or contextual module stacked on the segment-level representations to capture inter-utterance dependencies. We leave a systematic treatment of such streaming settings, together with their computational and real-time constraints, to future work.
